# Connectome-based biophysics models of Alzheimer’s disease diagnosis and prognosis

**DOI:** 10.1016/j.trsl.2022.08.008

**Published:** 2022-08-27

**Authors:** Justin Torok, Chaitali Anand, Parul Verma, Ashish Raj

**Affiliations:** a Department of Radiology, University of California, San Francisco, San Francisco, California; b Department of Bioengineering, University of California, Berkeley and University of California, San Francisco, Berkeley, California; c Department of Radiology, Weill Cornell Medicine, New York, New York

## Abstract

With the increasing prevalence of Alzheimer’s disease (AD) among aging populations and the limited therapeutic options available to slow or reverse its progression, the need has never been greater for improved diagnostic tools for identifying patients in the preclinical and prodomal phases of AD. Biophysics models of the connectome-based spread of amyloid-beta (A*β*) and microtubule-associated protein tau (*τ*) have enjoyed recent success as tools for predicting the time course of AD-related pathological changes. However, given the complex etiology of AD, which involves not only connectome-based spread of protein pathology but also the interactions of many molecular and cellular players over multiple spatiotemporal scales, more robust, complete biophysics models are needed to better understand AD pathophysiology and ultimately provide accurate patient-specific diagnoses and prognoses. Here we discuss several areas of active research in AD whose insights can be used to enhance the mathematical modeling of AD pathology as well as recent attempts at developing improved connectome-based biophysics models. These efforts toward a comprehensive yet parsimonious mathematical description of AD hold great promise for improving both the diagnosis of patients at risk for AD and our mechanistic understanding of how AD progresses.

## Overview

Alzheimer’s disease (AD) is an increasingly prevalent neurodegenerative disorder whose pathological hallmarks are abnormal deposits of amyloid-beta (A*β*) and microtubule-associated protein tau (*τ*). The buildup of aggregates of these proteins is progressive, irreversible, and associated with deficiencies in cognitive function and dementia. Both A*β* and *τ* exhibit characteristic spatiotemporal deposition patterns. The first A*β* plaques typically appear in temporobasal and frontomedial areas before spreading throughout the remaining neocortical areas and eventually the striatum.^1,2^ By contrast, *τ* tangles appear first in the locus coeruleus and then spread to the entorhinal cortex, hippocampus, temporal areas, and finally throughout the cortex.^1,3^ Both in vitro and in vivo evidence demonstrate that *τ* in particular predominantly migrates *trans-synaptically*, where white matter tracts between regions serve as the conduits for the transmission of *τ* from affected regions to unaffected regions.^4-7^ The staging of A*β* pathology also suggests that it spreads trans-synaptically,^8-10^ but passive diffusion in the extracellular space of the brain may also contribute.^11,12^ Understanding how the disease process unfolds over time is critical for ultimately finding effective treatments for AD, which has remained largely recalcitrant to pharmacological interventions despite decades of clinical trials.^13^

**Fig 1.**
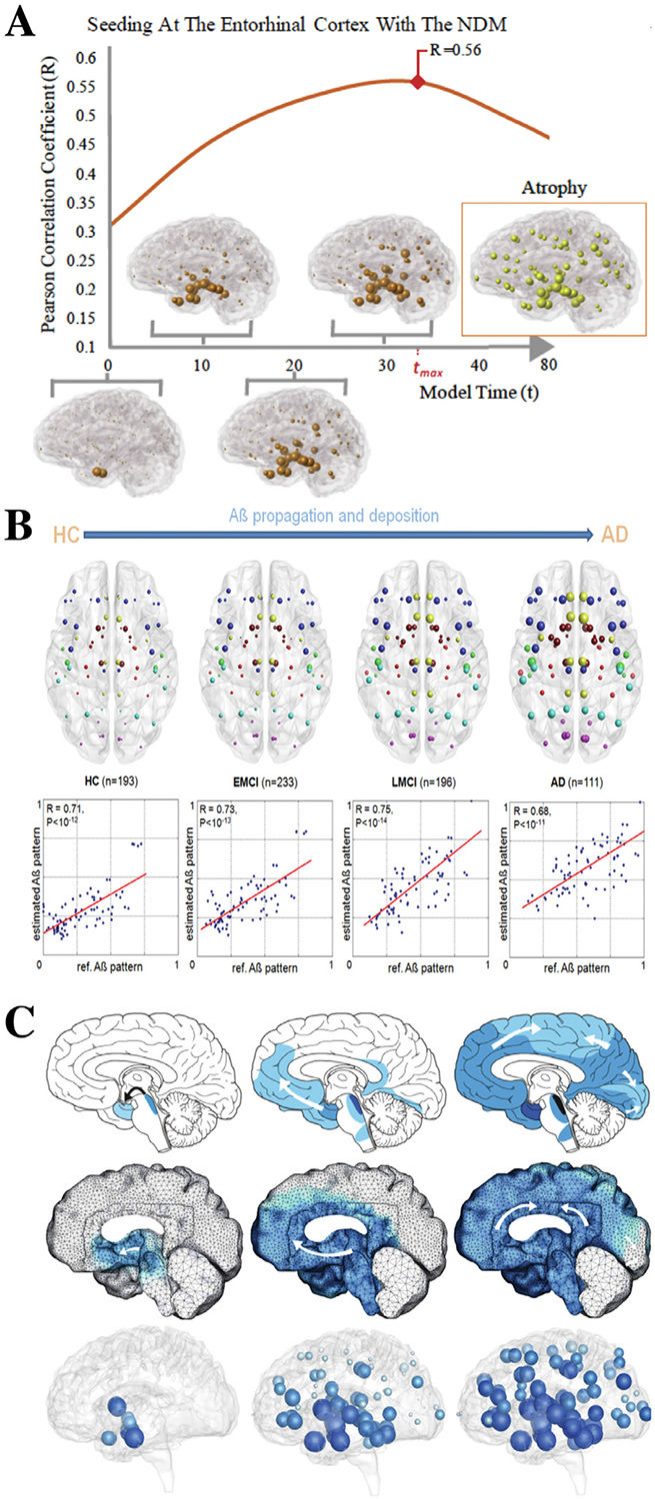
Survey of connectome-based spread models in AD. A: The NDM successfully recapitulated the spatiotemporal evolution of atrophy in AD in a patient cohort from ADNI.^20^ Starting from the entorhinal cortex, the model evolves with increasing atrophy following a network spread process, reaching a peak correlation at *t*_max_ with the ADNI pattern (inset). B, *Top*: PET-based mean regional A*β* deposition probabilities as modeled by the ESM in HC, EMCI, LMCI, and AD groups.^16^ Nodes correspond to 78 regions covering all the brain’s gray matter, with node sizes proportional to the associated A*β* burden. Modeled A*β* deposition progressively ramifies through brain circuits, starting mainly from the DMN regions to the rest of the brain. B, *Bottom*: Correspondence between the model and empirical PET regional A*β* deposition for the different groups. C: Classic Braak staging of AD tauopathy (*Top*) resembles both the continuum-space heterodimer model of tauopathy (*Middle*) and the Laplacian-based, network-spreading heterodimer model (*Bottom*) seeded at the transentorhinal and entorhinal regions.^15^ Abbreviations: A*β*, amyloid beta; ADNI, Alzheimer’s Disease Neuroimaging Initiative; EMCI, early mild cognitive impairment; HC, healthy controls; LMCI, late mild cognitive impairment; PET, positron emission tomography. (Reproduced from^24^).

Biophysical modeling of AD can provide a platform for integrating clinical and experimental data into a cohesive theoretical framework that also tests hypotheses for which direct evidence is difficult to acquire. The key insight for modeling trans-synaptic spread of a protein species is that it can be approximated by a *graph diffusion model*, where discrete gray-matter regions are the vertices of a graph and the structural connectivity values for all region pairs are its edge weights. More specifically, the Network Diffusion Model (NDM)^14^ and subsequent connectome-based spread models^15-19^ posit that, given an initial distribution of pathology in the brain, the regional pathology at future time points is a function of the concentration differences and connectivity values between all region pairs. Remarkably, despite the complexity of AD at a molecular and cellular level, these simple, macroscopic models recapitulate the canonical Braak staging of AD^18,19^ as well as pathology progression in human subjects.^17,20^ Not only that, but other disorders featuring the trans-synaptic spread of pathological protein species, such as Parkinson’s disease (PD)^21,22^ and amyotrophic lateral sclerosis (ALS),^23^, have also been modeled as a graph diffusion process with similarly strong results. Fig 1 summarizes key results from several connectome spread models.^24^ In addition to providing a tool for accurate, subject-specific predictions of where pathology will next appear, these models reinforce the validity of trans-synaptic spread as a fundamental feature of neurodegenerative disease, which has been controversial because it cannot be directly observed in patients.

While connectome-based spread models appear to capture an essential feature of the AD disease process, a more complete mathematical description of AD biology is necessary to gain further insights and improve the models’ predictive power. From a mathematical perspective, current models do not have sufficient complexity to explain how different protein species may spread to different sets of regions despite sharing the same point of initiation. In the case of *τ*, there is in vivo evidence that conformers with distinct microscopic properties exhibit diverse macroscopic deposition patterns when injected into the mouse hippocampus.^25^ Such a conformer-specific view of tauopathy has important implications for understanding not only what makes AD distinct from diseases such as frontotemporal lobar dementia (FTLD),^26-28^ but also the significant subject-to-subject heterogeneity among AD patients.^29-31^

Further, macroscopic connectome models of A*β* and *τ* spread have historically limited themselves to modeling the net effect of these proteins, regardless of their oligomeric diversity. In fact, it is well known that oligomers of varying sizes of both A*β* and *τ* remain in kinetic equilibrium, and these aggregation and fragmentation processes strongly contribute to their ability to spread throughout the brain.^32,33^ The modeling of the kinetics of protein aggregation are rather well established.^34-38^ Recently, a combined model of both aggregation and network transmission was proposed, which correctly recapitulated *τ* deposition patterns in AD patients.^39^ However, because it is currently not possible to characterize oligomeric diversity in vivo, it remains an open question how well these Smoluchowski-type models capture the complex interactions between oligomeric species in human disease. Given that it is smaller, soluble oligomers of A*β* and *τ* that are neurotoxic, not the larger, insoluble aggregates of these species,^40-45^ the understanding the equilibria between oligomers and aggregates is of keen interest.

Additionally, a necessary mathematical assumption endemic to connectome spread models is that all gray-matter regions have the same intrinsic properties, differing only in how they are connected to other regions. However, befitting their discrete biological functions, brain regions have distinct cytoarchitectures and neuronal compositions, some of which may be particularly susceptible to pathology.^46^ In addition to this selective neuronal vulnerability, support cells such as astrocytes^47,48^ and oligodendrocytes^49^ are known to participate in the AD disease process, and the microglia responsible for the neuroinflammatory response at sites of A*β* and *τ* deposition also strongly modulate the ability of these species to migrate to neighboring regions.^50-52^ Incorporating more features of AD biology at the level of the mathematical model while minimizing the risk of overfitting, particularly when regions are not treated identically, poses a significant challenge for enhancing current connectome-based spread approaches.

Here, we will expand upon several important facets of AD pathogenesis requiring further exploration from a mathematical modeling perspective:

1. Interactions between A*β* and *τ*
2. Asymmetric flows on the brain connectome
3. Neuroinflammation
4. Selective cell-type vulnerability
5. The relationship between neuronal activity and pathology

For each, we will provide biological context that justifies the need for model augmentation, related recent advances in mathematical modeling, and suggestions for next steps for modeling at the whole-brain level. Our aim is to synthesize the wealth of knowledge gained in disparate subfields of research towards a unified mathematical framework of AD that deepens our insight of the disease process and provides accurate, subject-specific predictions that can be used clinically for diagnosis, prognosis, and the tailoring of treatment.

## The clinical utility of mathematical models

A wide variety of tools have been applied to the problem of AD diagnosis. The primary method of diagnosing AD is through a battery of cognitive tests when the patient starts showing signs of dementia, such as the Mini Mental State Exam (MMSE)^53^ or the Clinical Dementia Rating (CDR).^54^ Although these exams are widely used and easy to administer, misdiagnosis of patients showing mild cognitive impairment (MCI) is still common, because their symptoms may not indicate that they will convert to AD.^55^ Accordingly, recent decades have seen significant developments in in vivo imaging techniques for detecting brain pathology directly. A*β*-sensitive positron emission tomography (PET) is currently in clinical use for the differential diagnosis of AD.^56,57^ Disruptions in glucose metabolism, as measured by 2-[^18^F]fluoro-2-Deoxy-D-glucose PET (FDG-PET), can also facilitate the early detection of AD.^57,58^ Although currently used only for research purposes, several PET radioligands for *τ* have also been developed, albeit with several off-target issues that remain to be solved.^59^ Structural magnetic resonance imaging (sMRI) is a less-expensive and minimally invasive alternative for detecting pathological changes in the brain, using techniques such as voxel-based morphometry (VBM) to determine regional gray-matter loss.^60^ Although the nonspecificity of brain atrophy as a biomarker has kept MRI-based atrophy detection from being the diagnostic tool of choice, several groups have been successful in using it to identify AD-related changes in structural brain features.^61,62^

To detect network-level changes in AD, diffusion tensor imaging (DTI), a special MRI sequence sensitive to the highly anisotropic diffusion of water within white-matter tracts, can map the structural connectome of the brain and identify disease-induced loss of white-matter integrity.^63^ Functional MRI (fMRI) is a complementary technique that can be used to detect AD-related changes in brain activity by the measuring the associated changes in blood oxygenation.^64^ Alternative technologies for investigating functional network dysfunction in AD include electroencephelography (EEG) and more recently magnetoencephelography (MEG), which detect brain activity with very high temporal resolution but lack the spatial resolution of fMRI. While these technologies are not in standard clinical use for AD diagnosis, they have been used to demonstrate abnormal oscillatory patterns in AD.^65-69^

Biophysical models can facilitate our understanding of these rich data in several key ways. Firstly, they can validate specific hypotheses about disease mechanisms that are difficult to observe directly in the clinic, often by linking disparate sources of data that are alone insufficient for explaining the disease process and cannot be combined in a purely statistical model. For instance, connectome spread models make concrete the mechanistic relationship between the brain connectome, determined by DTI, and brain-wide maps of imaging biomarkers measured by PET or sMRI.^14,16,18^ They can also provide subject-specific predictions of future pathology not yet observed, which can guide diagnosis and prognosis.^17,20^ As mentioned above, one of the most important challenges in AD diagnosis is to find a test that discriminates between patients with MCI who will later go on to develop AD and those who will not. Given that AD-specific treatments are unlikely to be effective in MCI patients who are not on the AD disease trajectory, a biophysical model with sufficient predictive power may be able to identify those patients who will benefit from therapies currently in clinical trials, leading to higher success rates.^70^ The identification of MCI-to-AD converters before the disease advances to the AD stage is also essential because the AD-associated loss of gray matter is effectively irreversible, so intervening prior to the loss of massive numbers of neurons is more likely to provide lasting, meaningful clinical benefits.^71^

The success of mathematical models of connectome-based spread allows exploration of another clinically relevant application: the possibility of “going back” in time to infer sites of pathology initiation that would best explain measured patterns of atrophy and pathology in a patient. For example, Torok et al. were able to “reverse” the NDM to infer the regional origins of pathology, called “seeds,” in individual patients on the AD spectrum.^30^ These patient-specific seeds, inferred by imposing a sparsity condition to constrain pathology to only a few regions, successfully reproduced known trends in AD and outperformed the best single-locus seed vector placed in the hippocampus. Subsequently, Maia et al. implemented a similar approach to infer seeds in PD, finding that PD patients could be grouped into two distinct clusters based on their seed patterns that exhibited markedly different ages of onset.^72^ These approaches enforced a single seeding event; however, a related method allowing several seeding events up to the observation window also produced plausible prior states of pathology.^73^ Therefore, biophysical models can give a glimpse into preclinical disease states that may be particularly useful for diagnosis.

Lastly, the analysis of sufficiently rich biophysical models can reveal which processes control disease dynamics, thereby indicating key mechanisms that future treatments should be designed to disrupt. While further work with experimental models of AD and the continued development of methodological tools for studying AD in the clinic are also needed to fully realize all of these goals, we can still advance the state of AD mathematical modeling by incorporating more biological detail, as we expand upon in the following sections.

## Interactions between A*β* and *τ* define AD pathogenesis

The prevailing view guiding the study of AD pathogenesis is the *amyloid cascade hypothesis*^74,75^: From a complex interaction of genetic predispositions and environmental triggers, aberrant cleavage products of amyloid precursor protein (APP) aggregate to form A*β* plaques. At a later time, abnormal deposits of *τ* known as neurofibrillary tangles (NFTs) form, a process which is facilitated in part by A*β*. Once the ability of the brain to clear these pathological accumulations is exhausted, misfolded assemblies of *τ* spread throughout the brain along white-matter tracts, eventually leading to neuronal death and irreversible, progressive cognitive decline. However, several pieces of evidence complicate the picture above. Perhaps most importantly, cognitively normal individuals who are amyloid-positive (A*β*+) exhibit at most modest deficits compared to age-matched amyloid-negative (A*β*−) individuals.^76^ Conversely, a percentage of patients clinically diagnosed with AD exhibit *τ* but not A*β* pathology^77^; moreover, *τ* misfolding and propagation causes a wide array of neurodegenerative disorders, albeit with distinct deposition patterns and clinical features from AD.^78^ Additionally, A*β* plaques and *τ* tangles poorly colocalize in the brains of AD patients at early stages of disease, making it challenging to understand how and when they interact to give rise to what is thought to be a canonical staging of neuropathology. There is evidence suggesting that A*β* and *τ* may interact at neuronal synapses and contribute to synaptic dysfunction.^79,80^ Further, A*β* may promote *τ* misfolding and aggregation through a cross-seeding mechanism.^81,82^ The interactions between A*β* and *τ* also need not be direct. For instance, the presence of amyloid particles in the extracellular space may trigger a cellular response that induces or facilitates the misfolding and aggregation of *τ*.^83^ Nevertheless, studying either A*β* or *τ* in isolation gives an incomplete picture of AD pathogenesis, and a necessary extension going forward is to jointly model these two types of pathological aggregates.

The foundational work in the biophysical modeling of AD pathology at the macroscopic level has focused on single-species models: for instance, *τ* in the case of the ND^20,30^ and Fisher-Kolmogorov (FK) models, ^17-19,84^ and A*β* in the case of the Epidemic Spread Model (ESM).^16^ However, more recent work has turned towards combined A*β* – *τ* models at various spatial scales. Kuznetsov and Kuznetsov, following up on prior work solely modeling *τ*,^85^ modeled the effect of A*β* concentrations on the production and migration of *τ* in the axons of diseased neurons, where A*β* increased the autocatalysis of *τ* aggregation.^86^ Their microscopic kinetic model was capable of fitting the distribution of axonal *τ* in an *in vitro* cell culture system.^87^ Another valuable advance came from Bertsch et al., who simulated a multi-species model of A*β* and *τ* under conditions of purely isotropic diffusion.^88^ In this system, they found that A*β* agglomeration rate controlled much of the dynamics of the system. Interestingly, only *sufficiently small* agglomeration rates were able to result in widespread brain malfunction, because the irreversible formation of nontoxic A*β* plaques in their model needed to be slow enough for the toxic oligomeric species to diffuse. Further, the addition of *τ* to the model resulted in more severe, widespread disease relative to its A*β*-only counterpart, reinforcing the notion that the true dynamics of brain malfunctioning only emerge when jointly modeling both A*β* and *τ*. These microscopic and mesoscopic models motivate the development of network-level interaction models that can be fit to clinical data.

At the macroscopic level, too, there have been advances towards an integrated A*β* – *τ* mathematical model. Raj and Anand sought to model both protein species together by adding an A*β* – *τ* interaction term that induces *τ* expansion in brain regions where they co-localize, in addition to the connectome-based spread of both species.^89^ This minimalist approach reproduced the key features of the spread of both A*β* and *τ* in the brain as determined by PET imaging of MCI and AD patients. On the maximalist side, Bertsch et al., following up on their previous work discussed above,^90^ proposed a more general framework for exploring A*β* and *τ* dynamics at multiple spatiotemporal scales that includes mathematical treatments of: oligomer size dependence, the role of the lymphatic system, the secretion and reuptake of toxic species, neuronal dysfunction, and microglial activation in response to pathology (see Section Modeling interactions between pathology and the immune response below). We refer the reader to their work for the mathematical details of their highly detailed proposal.

The approaches above illustrate the central tension in terms of biophysically modeling not only A*β* – *τ* interactions but all extensions to the network model: defining the simplest model that sufficiently captures the relevant features AD biology and does not compromise on its predictive capacity. On the one hand, the Raj and Anand model reduced the dynamics of A*β* and *τ* to a single term.^89^ While they demonstrated the predictive value of adding this term, questions remain about whether a more complex model incorporating more of the direct and indirect interactions of these two species could provide significantly more accurate predictions, which will be necessary for diagnosis and prognosis in the clinic. On the other, the model of Bertsch et al. requires significant exploration from a parameter sensitivity perspective to be useful as a predictive model.^90^ More specifically, to reduce the risk of overfitting, detailed analysis will be necessary to exclude features that are mathematically redundant or add little to the richness of the dynamics of the system. Nevertheless, these approaches represent an initial step towards a robust and clinically useful description of A*β* and *τ* interactions.

## Directionality of specific conformers parsimoniously explains diverging *τ* deposition patterns

In addition to posing a challenge diagnostically for cases with mixed clinical presentations, how the misfolding of *τ* can give rise to such disorders as AD, FTLD, Pick’s disease, and corticobasal degeneration, among others, is an unresolved question. A parsimonious explanation is that each of these tauopathies has characteristic *conformers* of *τ*, and it is the differences in microscopic properties between these conformers that largely controls the rate of progression and the spatiotemporal patterns of *τ* deposition.^78^ The evidence for this conformation-specific hypothesis of tauopathy comes from varied sources. Direct examination of the structure of *τ* aggregates using electron microscopy has revealed that *τ* aggregates from different sources adopt distinct conformations.^91,92^ Synthetic *τ* fibrils coaxed to misfold and aggregate under various in vitro conditions exhibit different molecular properties,^93^ and the injection of different fibrils into the brains of mice results in different patterns of *τ* deposition.^25,94^ Clinically, the recent discovery of AD-specific phosphoepitopes of *τ* in the CSF and bloodstream that discriminate between patients with AD and patients with primary tauopathies with very high accuracy lends further credibility to the notion that *τ*-aggregate structure is a key factor in human disease.^26-28^ Microscopic properties of *τ* isolates from the brains of AD patients could also be tied to differences in their clinical presentations.^29^ However, how distinct conformers of *τ* give rise to different clinical phenotypes is poorly understood.

One of the mechanistic underpinnings of *τ*-conformer-specific deposition patterns may be that each species exhibits a different *directional spread bias*. In other words, *τ* may not migrate in a purely *diffusive* manner along concentration gradients, as is commonly assumed in connectome-based spread models, but instead spreads preferentially in the *anterograde* (ie, with axon polarity) or *retrograde* (ie, against axon polarity) directions. Biophysically, both healthy and pathological *τ* variants may freely diffuse or be transported by molecular motors attached to microtubules, giving rise to asymmetric *τ* distributions in the neuron.^95^ The hyperphosphorylation of pathological *τ* disrupts its direct interactions with microtubules^96,97^ and the motor proteins themselves^98-100^; together, these effects lead to aberrant axonal transport and the mis-sorting of *τ* into the neuronal somatodendritic compartment.^101,102^ While axonal transport and diffusion occur on a much faster timescale than the detectable pathological changes during AD,^95,103^ the establishment of persistent directional biases vastly influences how *τ* ultimately propagates along the brain connectome.

To explore the phenomenon of *τ* axonal transport in the disease state at a microscopic level, Torok et al. developed a two-species, multicompartment model to explore the interactions between the pathological axonal transport of *τ* and the formation and breakdown of insoluble *τ* aggregates.^104^ Leveraging insights from in vitro work demonstrating that the primary anterograde-directed motor protein, kinesin-1, has increased activity in the presence of monomeric hyperphosphorylated *τ*^98-100^ and is knocked down by *τ* aggregates,^105^ the mathematical model poses a simple *τ*-concentration-dependent feedback mechanism on the anterograde velocity of *τ* transport. The authors interrogated the complex dynamics that emerge between the interplay of *τ* aggregation and axonal transport feedback, finding that higher aggregation rates generally led to stronger retrograde biases in *τ* deposition at steady-state. Although further experiments and more detailed modeling work are warranted, this work represents one of the first attempts to connect the microscopic properties of *τ* conformers to pathological changes that can be observed at macroscopic timescales.

**Fig 2.**
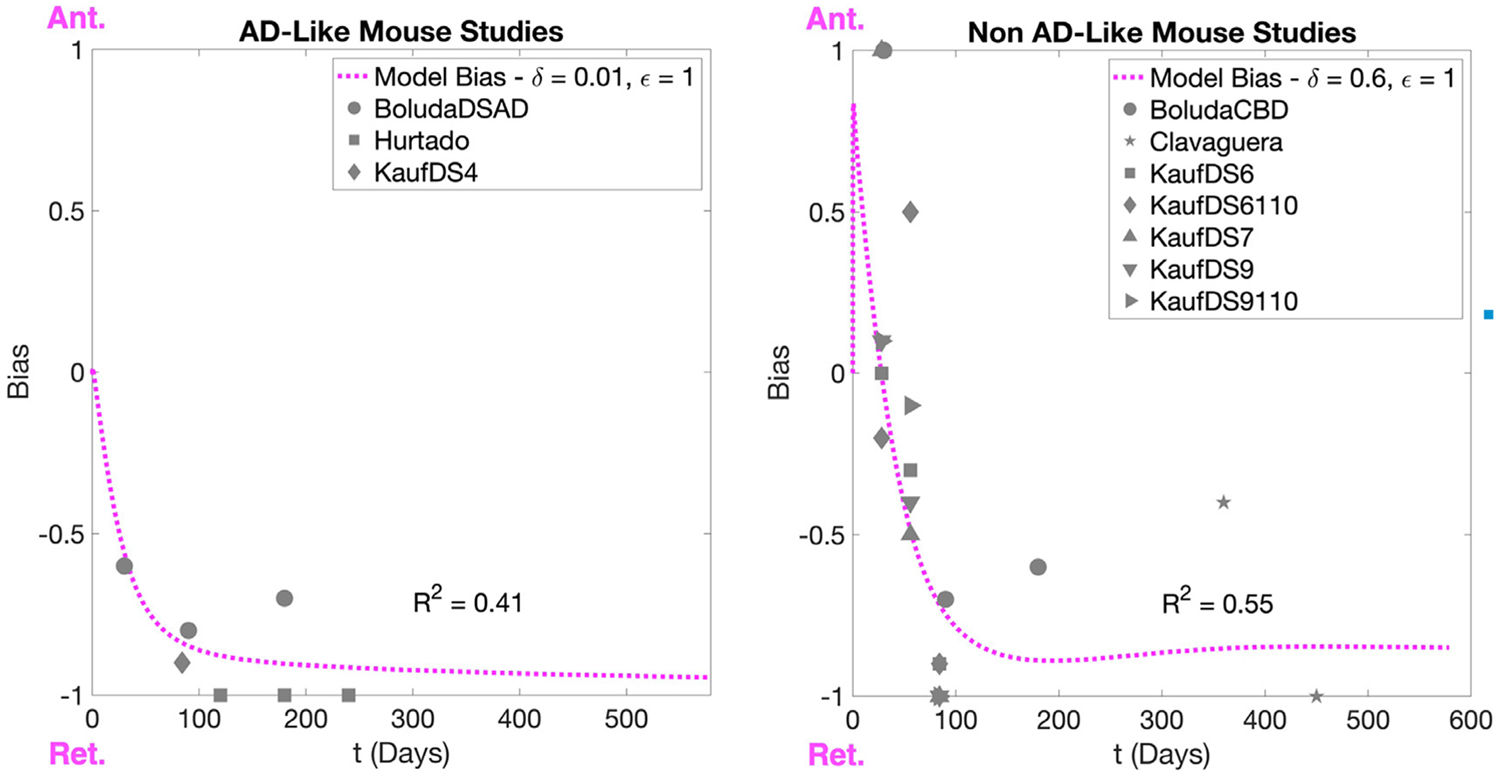
Transport vs. network directional bias in mouse models of tauopathy. The network directional biases for a collection of tauopathy models, separated by whether the *τ* strains involved misfolded in the presence of A*β* or not,^106^ were fit with using an axonal transport model including *τ*-kinesin interactions. The agreement between these two assessments of bias indicates that the interplay of axonal transport regulation and *τ* aggregation and fragmentation processes on a microscopic level can explain macroscopic directional biases. The mouse tauopathy data come from the following studies: “BoludaCBD”, “BoludaDSAD’^177^; “Hurtado’^178^; “Clavaguera’^4^; “KaufDS4”, “KaufDS6”, “KaufDS6110”, “KaufDS7”, “KaufDS9”, “KaufDS9110.”^25^ (Reproduced from^104^).

Directionally biased spread has also been explored at the whole-brain level. Mezias et al. explored the role of directionality using *τ* quantification in fifteen mouse models of tauopathy,^106^ utilizing the Allen Mouse Brain Connectivity Atlas (AMBCA) from the Allen Institute for Brain Science (AIBS), which not only provides connection strengths between region-pairs but also the directionality of those connections.^107^ By introducing a bias parameter, they were able to demonstrate that *τ* fibrils formed in the presence of A*β* exhibit a more pronounced retrograde bias than A*β*-naive *τ*, an effect which may be attributed to higher *τ* aggregation rates in the Torok et al. framework discussed above.^104^ Further, the temporal progression of directional bias as modeled by the axonal transport model could be parameterized to fit the network directional bias for a variety of mouse models (Fig 2). Given that significant in vitro evidence exists that A*β* indeed induces *τ* aggregation and the formation of unique *τ* conformations, the transport feedback mechanism parsimoniously explains divergence in *τ* deposition patterns in the context of connectome-based spread. Directional NDM models have also demonstrated that *α*-synuclein preferentially migrates in the retrograde direction in mouse models of PD,^108,109^ suggesting that transport-mediated directional biases may be a common feature of prion-like neurodegenerative diseases.

Several challenges remain before we can fully integrate directional spread bias into connectome-based models of human disease. For one, rather than posing the network directional bias parameter phenomenologically, as has been done previously, it should be made a function of the axonal transport parameters and aggregation rate. In this way, the linkage between microscopic *τ* properties and macroscopic directional bias is made explicit. Axonal transport dysregulation may also be mediated by other interactions that have not yet been considered. For instance, a joint A*β* – *τ* model may be required to fully capture these dynamics, since there is evidence that A*β* impacts axonal transport in a *τ*-independent manner,^110^ dovetailing into the other considerations discussed in Section Interactions between A*β* and *τ* define AD pathogenesis. Another issue is that any mathematical description of directionally biased spread requires that the brain connectome be a *directed*, asymmetric graph that distinguishes afferents from efferents. However, DTI, the only technique capable of quantifying inter-regional connectivity in vivo, cannot resolve brain connectivity at this level and is definitionally *undirected*. One way to circumvent this issue is to infer the proportion of afferents to efferents in the human connectome by examining homologous brain structures in the AMBCA^107^ and the macaque connectivity atlas (CoCoMac),^111^ which were obtained using viral tracing. While the assumption of homology will not hold across all connections, limiting the degree to which connection polarity can be completely determined, such a cross-species connectome should be sufficient for a preliminary exploration into fitting connectome-based spread models with directionality to clinical data. As a first demonstration, retrograde modes of transmission along a cross-species connectome were shown to produce the best-fitting connectome-based spread model for brain pathology in progressive supranuclear palsy (PSP) patients.^112^ The development of effective imaging techniques for reconstructing single-cell-level resolution projections in postmortem human brain tissue, such as FAST^113^ and SHANEL,^114^ indicates that significant progress is underway in producing a fully directed, high-resolution human connectome, which will advance biophysical modeling in humans tremendously. Overall, the relaxation of the “diffusive spread” assumption in connectome-based models may represent an avenue to greatly increasing their accuracy without dramatically increasing their complexity.

## Modeling interactions between pathology and the immune response

Discussion in the previous sections has been concerned with the mathematical modeling of A*β* and *τ* without any consideration of region-intrinsic properties. However, the presence of these pathological species effects a strong local response from the neuroimmune system, which is primarily mediated by microglia, the resident immune cells of the brain. Whether this response is protective or harmful appears to be context-dependent. Early in the disease process, microglia effectively clear A*β* pathology in mouse models,^115,116^ but over time, their capacity to remove plaques is attenuated.^117^ Additionally, the neuroinflammatory response mounted by microglia in response to A*β* induces neurodegeneration; suppression of microglia in 5xFAD mice prevented hippocampal neuronal loss.^118^ With regards to *τ*, too, the neuroimmune response is complex and multifaceted. Gliosis, the expansion of microglial populations as part of the immune response, was found to correlate with AD disease severity, both in terms of clinical symptoms and *τ* burden.^119^ Activated microglia have also been shown to internalize *τ* aggregates, and were discovered in a postmortem examination of the brains of AD patients.^120^ While microglia have shown some capacity to clear *τ* pathology,^121,122^ their depletion in mouse models resulted in reduced *τ* propagation^123^ and neurodegeneration.^124^ Although the above picture is incomplete, the microglial response is tightly coupled to both the emergence and progression of AD pathology.

Of particular importance is the role of disease-associated microglia (DAM), a unique subtype of microglia identified from human AD brain samples and 5xFAD mouse models. These glia are characterized by a unique gene expression profile with elevated expression of certain AD-related genes and lower expression of homeostasis microglial genes.^125^ The DAM response is mediated by the microglial TREM2 receptor, which induces a neuroprotective, phagocytic response to A*β*^126^; indeed, rare loss-of-function variants of the *TREM2* allele confer an increased risk for AD and other dementias.^127-129^ How neuroprotective DAM are in ameliorating A*β* pathology also may be disease-stage-dependent: in a mouse amyloid model, *TREM2* deficiency was associated with decreased plaque number and area early in disease but increased plaque size and area late in disease.^130^ Interestingly, a mouse tauopathy model expressing human TREM2^R47H^, a variant associated with increase AD risk, exhibited reduced *τ* pathology and neurodegeneration, suggesting that the DAM response is harmful with respect to *τ*.^131^ *τ* pathology itself is also capable inducing an inflammatory microglial response that was associated with synaptic loss.^132^ Recently, a PET imaging study in cognitively normal, MCI, and AD subjects revealed that microglial activation, which was associated with *TREM2* expression, followed Braak staging alongside *τ* pathology, and that microglial activation networks were more strongly associated with longitudinal *τ* progression.^133^ Pascoal et al. also found that A*β* enhances microglia-activation-associated *τ* spreading, suggesting a role for activated microglia as mediators of the deleterious effects of A*β* on *τ* pathology.^133^

**Fig 3.**
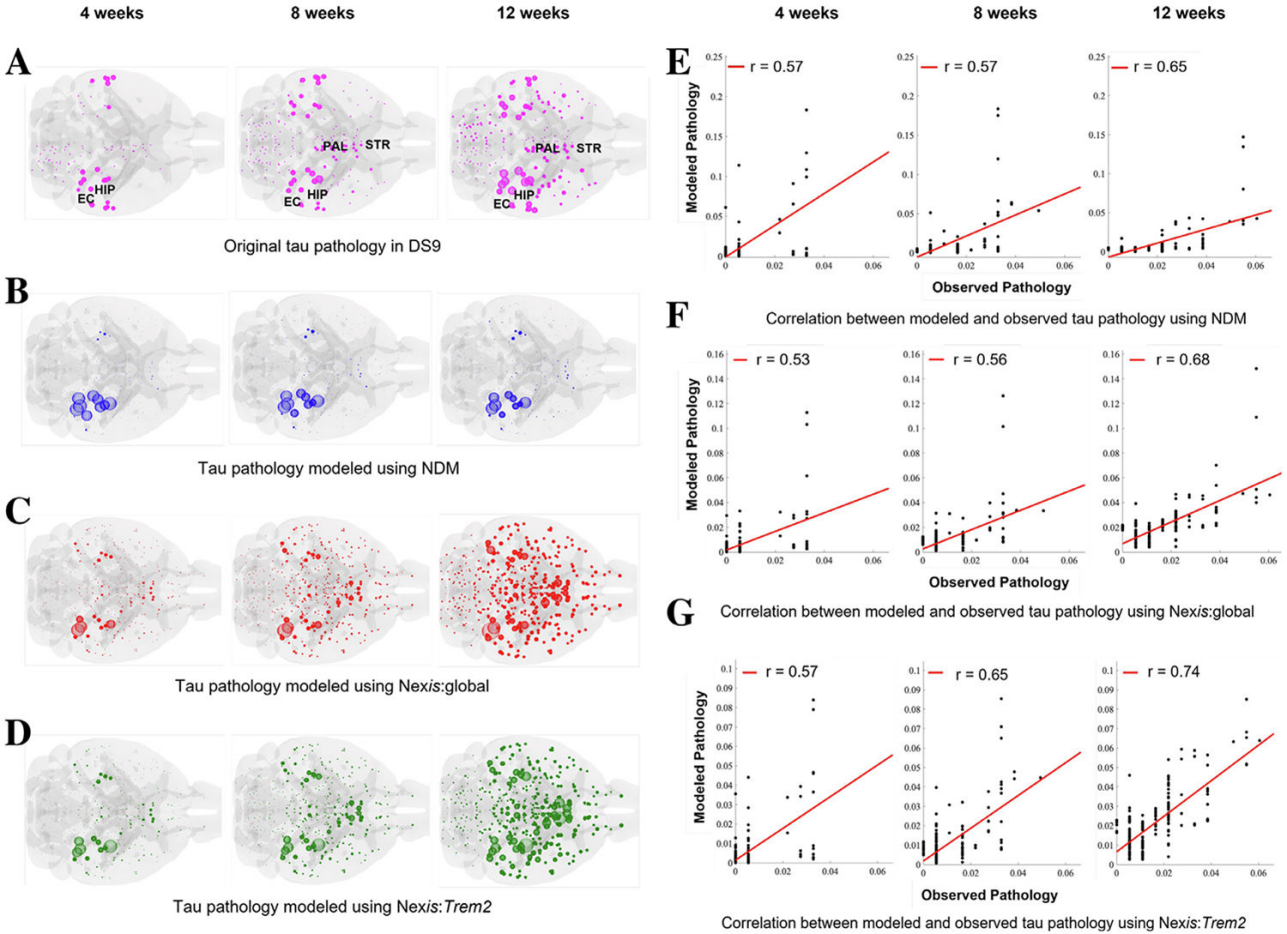
Connectome-based model incorporating *Trem2* provides a superior fit of mouse tauopathy. A: Glass-brain rendering of the *τ* pathology in DS9-injected mice^25^ predominantly affects the entorhinal cortices (EC) and hippocampus (HIP) at early time points, then spreads throughout the neocortex, pallidal (PAL), and striatal (STR) areas. B: Glass-brain rendering of the *τ* pathology modeled using the Network Diffusion Model (NDM).^14^ C: Glass-brain rendering of the *τ* pathology modeled using the Nexopathy *in silico* model, which augments the NDM model with a term modeling the accumulation of *τ* pathology intra-regionally without incorporating effects from inflammation marker genes (Nex*IS*:global). D: Glass-brain rendering of the *τ* pathology modeled using the Nex*IS* model with *Trem2*-dependent modulation of *τ* accumulation and transmissibility (Nex*IS:Trem2*). E: The per-time-point fits of the NDM are moderately strong, with R^2^ = 0.25 over all time points. F: The per-time-point fits of Nex*IS*:global are similar to that of the NDM (E). However, because the addition of an accumulation term allows the model to account for the amplification of DS9 *τ* pathology over time (A) – note the difference in the scale of the y-axes between (E) & (F) – the overall fit across all time points is much stronger (R^2^ = 0.44). G: Nex*IS:Trem2* provides the best overall model for DS9 pathology, both in terms of per-time-point and overall fits (R^2^ = 0.52). (Reproduced from^134^).

Given the complex roles of the neuroimmune response in both remediating and potentiating AD-related pathological changes, the development of a mathematical model that can account for these different effects would yield greater insights into the disease process. Bertsch et al. recently proposed to model activated microglial populations as a function of A*β* and *τ* concentrations, with the latter depending in part upon activated-microglia-dependent clearance.^90^ Further, Anand et al. augmented the original NDM approach^14^ by incorporating gene-expression-dependent accumulation and transmissibility parameters.^134^ The resulting modeling platform, called Nexopathy in silico (Nex*IS*) following the paradigm proposed by Warren et al.,^135^ was able to demonstrate that a connectome-based model including of baseline *TREM2* expression was significantly more predictive of *τ* pathology progression in mouse tauopathy models^25^ than models including no gene expression information (Fig 3). Further, the values of the *TREM2*-associated accumulation and transmissibility parameters suggested that activated microglia are associated with a local reduction in *τ* accumulation rate, in agreement with observations of their ability to internalize and degrade *τ*,^121,122^ but also with increased transmissibility, supporting a role for activated microglia in potentiating *τ* spreading.^123^ These results indicate that connectome-based spread models can be used to validate experimental observations at a whole-brain level.

Future efforts should strive to directly model the populations of activated microglia in AD patients in addition to A*β* and *τ* pathology. One limitation of the Nex*IS* approach is that it relied solely on baseline *TREM2* expression data because the changes whole-brain distribution of *TREM2* expression levels over time in mouse tauopathy models are poorly characterized. However, a dynamic model of microglia activation could be constructed following the suggestion of Bertsch et al.,^90^ with an initial configuration given by baseline *TREM2* expression. Particularly intriguing is the suggestion that there is a temporal staging of pathological events in AD with activated microglia as mediators of A*β*-induced *τ* pathology^133^; here, connectome-based biophysical models of all three populations could uncover how they interact at a mechanistic level. Following the Nex*IS* model comparison approach, models of varying complexity could be tested against each other to determine the key biophysical interactions that are necessary for achieving strong fits to imaging data. In this way, connectome-based biophysical models can be used to probe biological hypotheses about the roles of microglia in AD pathophysiology, which are still only partly understood.

## Reconciling cell-type vulnerability and connectome-based spread

Another factor thought to contribute to the regional specificity of neurodegeneration in AD is *selective neuronal vulnerability*: the neuronal composition of strongly affected regions, such as the hippocampus and entorhinal cortex, confers to them a higher innate susceptibility to AD pathology.^46^ With the advent of technologies such as single-cell RNA sequencing (scRNAseq) and single-nucleus RNA sequencing (snRNAseq), it has been established that the transcriptomic programming of the brain is altered in AD in a cell-type-specific manner.^136,137^ Recent work identifying subtypes of excitatory neurons that appear to be particularly impacted by *τ* pathology, both in AD^138,139^ and FTLD,^140,141^ indicates that selective vulnerability may indeed play an important role in the disease process. We note here that selective vulnerability is not in opposition to trans-neuronal spread as a disease mechanism and may not be entirely separable from it, because many of the vulnerable neuronal subtypes have long-range projections. Insofar as they are separable, however, it is likely that both cell-autonomous and trans-neuronal mechanisms contribute to the complex pathophysiology of AD.

The isolation and transcriptomic characterization of neuronal subtypes that are particularly susceptible to AD pathology has been limited to brain subregions that are canonically affected early in the disease process, such as layer II of the entorhinal cortex.^139^ A broader investigation of the influence of regional cell-type composition on vulnerability to pathology, however, requires the mapping of a comprehensive set of cell types across the whole brain. Kim et al. recently quantified the distributions of *Pvalb*-, *Sst*-, and *Vip*-expressing *γ*-aminobutyric acidergic (GABAergic) interneurons in the mouse brain,^142^ and Li et al. similarly mapped the mouse cholinergic system.^143^ Developments in in situ transcriptomics have facilitated the complete cellular characterization of specific regions of the mouse brain,^144-146^ but these technologies have not yet been scaled up to the entire cortex. More recently, a computational technique called Matrix Inversion and Subset Selection (MISS)^147^ produced highly accurate maps of cell types by using their transcriptomic signatures, as determined by single-cell RNA sequencing (scRNA-seq),^148-150^ to deconvolve the voxelwise gene expression data contained in the Allen Gene Expression Atlas (AGEA).^151^ The breadth of mapped neuronal and non-neuronal cell types coupled with their whole-brain spatial coverage makes the MISS-inferred distributions in particular highly useful for an investigation into selective vulnerability.

From a biological perspective, there are several ways in which cell types may play a role in mediating the pathological spread of A*β* and *τ*, which directly inform how traditional models of connectome-based spread should be modified. A high density of neuronal cell types that are especially susceptible to proteinopathy in a given region may effectively increase the local accumulation rates of A*β* and *τ*; conversely, neuroprotective cell types, particularly certain support cells, may decrease these accumulation rates by enhancing clearance at a regional level. Another possibility is that the presence of certain cell types may modulate regional transmission rates. For instance A*β* is known to be cytotoxic to oligodendrocytes, the primary support cells responsible for the maintenance of the myelin sheath around long-range axons.^152,153^ Oligodendrocytes have also been observed to internalize *τ* as well as facilitate its seeding and propagation.^47^ Astrocytes, which regulate blood flow and support healthy neuronal function,^154,155^ can synthesize and secrete A*β*, thereby contributing to overall plaque burden.^156,157^ They can also internalize and degrade *τ*, although when their clearance mechanisms fail, they serve as reservoirs of toxic oligomers that can be re-released and propagate to neighboring neurons.^158^ Therefore, we posit that the Nex*IS* framework described above^134^ (see Section Modeling interactions between pathology and the immune response), when incorporating MISS-inferred cell-type densities instead of the regional expression of immune response genes, provides a low-complexity modeling platform that jointly describes selective cell-type vulnerability and network spread. It is worth emphasizing that without pre-established cell-type distributions, a joint model would require multiple independent parameters per region in addition to the global parameters normally considered in network spread models, which quickly runs into issues of overfitting. In addition to providing quantitatively better models of AD, this approach enables hypothesis-based model comparison between the original network spread model and the joint model including cell types of interest, which can be used to uncover new interactions between cell types and pathology spread.

While the Nex*IS* approach has only been applied on regional *τ* pathology in mouse models, there are no mathematical limitations to applying it to tau-PET imaging data from AD patients in combination with the Allen Human Brain Atlas (AHBA)^159^ as discussed above (see Section Modeling interactions between pathology and the immune response). However, currently there is a data-based limitation to Nex*IS* with selective cell-type vulnerability: the MISS pipeline has thus far only inferred cell-type maps in the mouse brain and no equivalent maps exist for human brain. While it the lacks the spatial resolution of the AGEA, MISS can still leverage the AHBA and publicly available human brain scRNAseq data (ie, from the AIBS^160^) to infer regional distributions of human neuronal and non-neuronal cell types, which can then be used within the Nex*IS* framework. Another consideration is the impact of neurodegeneration itself; that is, the dynamicity of regional cell-type densities over the course of disease. Other models have considered a one-way interaction where the presence of A*β* or *τ* pathology induces atrophy,^18,90^ but since Nex*IS* posits that local cell-type densities influence how pathology accumulates and spreads, neurodegeneration over time should also feed back into the evolution of A*β* and *τ* deposition. This suggests that Nex*IS* be augmented to also describe atrophy, which should be straightforward from both mathematical-modeling and data-collection perspectives, since the T1-weighted MRI images used to assess regional volume loss (see Section The clinical utility of mathematical models) are often collected in tandem with PET imaging in large clinical datasets, such as the Alzheimer’s Disease Neuroimaging Initiative (ADNI).^161^ Overall, until recently it has not been possible to reconcile the impact of selective neuronal vulnerability in the context of connectome-based spread at a whole-brain level, and the MISS and Nex*IS* platforms provide a promising avenue for exploring these two hypotheses together.

## Functional deficits in AD facilitate the disease process

Evidence indicates that connectome-based spread of A*β* and *τ* pathology can be facilitated by abnormalities in structural or functional connectomes, but the contribution of each is the subject of ongoing research.^14,162-166^ Raj, 2021^24^ argues that while it is possible that pathology spread follows functional network dysfunction^163^ it is also possible that pathology is indeed driven by the structural connectome, and the association between pathology spread and functional network is a consequence of the strong coupling of functional and structural connectomes. ^167,168^ Even though the role of functional network deficits in pathology spread is unclear, cellular-level alterations in excitatory-inhibitory balance are also associated with A*β* and *τ*.^169-173^ Particularly, basic science studies indicate that A*β* and *τ* are associated with neuronal hyper- and hypoactivity, respectively.^170^ There is also evidence suggesting that neuronal action potentials stimulate the production of A*β*^174^ and the release of endogenous *τ*,^175^ implicating a direct role for functional activity and the progression of protein-based pathologies. Together, the interactions between structural connectivity, functional connectivity, A*β*, and *τ* have significant roles in the evolution of AD-related brain dysfunction.

Recently, these interactions were partly elucidated by a study combining MEG, PET, and biophysical modeling.^176^ In this work, the authors reported altered excitatory and inhibitory regional model parameters in AD. In particular, an excitatory model parameter was uniquely associated with *τ* deposition, partially mediating the altered oscillatory activity in the *α* frequency band. By contrast, the inhibitory model parameter was uniquely associated with A*β* deposition, partially mediating altered oscillatory activity in the *δ*-*θ* frequency band. This study opens up new directions of identifying cellular-level mechanisms in AD using biophysical modeling and multimodel imaging. More specifically, modeling the connectome-based spread of A*β* and *τ* and their interactions at a network level (see Section Interactions between A*β* and *τ* define AD pathogenesis), coupled with a mathematical description of how these species interact with the functional network in AD, could uncover novel mechanisms of clinical importance. Further, such a model could be augmented by incorporating region-intrinsic properties, such as densities of specific subpopulations of excitatory and inhibitory neurons (See Section Reconciling cell-type vulnerability and connectome-based spread). While previous modeling approaches have included a treatment of neuronal death as a function of A*β* and *τ* deposition,^18,90^ this coupling of protein pathology and functional abnormalities in AD is relatively unexplored and therefore presents a unique opportunity to interrogate their relationship at a whole-brain level.

## Concluding thoughts

The directions for connectome-based biophysics models discussed above are by no means an exhaustive list, and as further insights are made into AD pathophysiology, mathematical models should be augmented to accommodate them. Pursuing each avenue of investigation on its own will likely yield at least incremental improvements in the network models’ predictive capacity, as has already been demonstrated in several cases.^89,106,134^ Further, the insights gained from more inclusive and robust models have the potential to identify key mechanistic interactions with translational impact. Moving forward, the field should move towards more complete biophysical descriptions of AD pathophysiology while maintaining parsimony where possible, given the risk of overfitting to pathology data that may be lacking in temporal resolution, spatial resolution, or both. We anticipate that as mathematical models continue to improve, the area of intersection between the diagnosis and prognosis AD in patients and biophysics-based modeling of the underlying pathology will only continue to grow.
